# Xenoestrogens Alter Estrogen Receptor (ER) α Intracellular Levels

**DOI:** 10.1371/journal.pone.0088961

**Published:** 2014-02-20

**Authors:** Piergiorgio La Rosa, Marco Pellegrini, Pierangela Totta, Filippo Acconcia, Maria Marino

**Affiliations:** Department of Science, University Roma Tre, Rome, Italy; University of Naples 2, Italy

## Abstract

17β-estradiol (E2)-dependent estrogen receptor (ER) α intracellular concentration is a well recognized critical step in the pleiotropic effects elicited by E2 in several target tissues. Beside E2, a class of synthetic and plant-derived chemicals collectively named endocrine disruptors (EDs) or xenoestrogens bind to and modify both nuclear and extra-nuclear ERα activities. However, at the present no information is available on the ability of EDs to hamper ERα intracellular concentration. Here, the effects of bisphenol A (BPA) and naringenin (Nar), prototypes of synthetic and plant-derived ERα ligands, have been evaluated on ERα levels in MCF-7 cells. Both EDs mimic E2 in triggering ERα Ser118 phosphorylation and gene transcription. However, only E2 or BPA induce an increase of cell proliferation; whereas 24 hrs after Nar stimulation a dose-dependent decrease in cell number is reported. E2 or BPA treatment reduces ERα protein and mRNA levels after 24 hrs. Contrarily, Nar stimulation does not alter ERα content but reduces ERα mRNA levels like other ligands. Co-stimulation experiments indicate that 48 hrs of Nar treatment prevents the E2-induced ERα degradation and hijacks the physiological ability of E2:ERα complex to regulate gene transcription. Mechanistically, Nar induces ERα protein accumulation by preventing proteasomal receptor degradation via persistent activation of p38/MAPK pathway. As a whole these data demonstrate that ERα intracellular concentration is an important target through which EDs hamper the hormonal milieu of E2 target cells driving cells to different outcomes or mimicking E2 even in the absence of the hormone.

## Introduction

17β-estradiol (E2), the most active estrogen, exerts profound effects on the growth, differentiation, and functioning of many reproductive and non-reproductive tissues. E2 determines its pleiotropic actions by binding to the nuclear estrogen receptor (ER) α and β, which act as ligand-activated transcription factors regulating the transcription of the estrogen response element (ERE) containing genes. In addition to these ER nuclear actions, E2 is also able to elicit the rapid activation of a plethora of extra-nuclear signalling pathways by virtue of engaging the membrane-localized receptors. Integration of nuclear and extra-nuclear ER-dependent actions as well as of ERα and ERβ specific signalling co-ordinately contributes to the regulation of the E2 physiological actions [1], [2].

As per other hormones, all the E2 effects occur in parallel with transcriptional and post-transcriptional modulation of ER intracellular concentrations, which are finely modulated by E2-induced extra-nuclear [3], [4] and epigenetic signalling (e.g., ER promoter methylation, microRNAs, miRNAs) [5]. As an example, the relative concentration of ERα and ERβ is significantly altered during the development of breast cancer with an increase in ERα levels and a decrease in ERβ concentration [6]. Moreover, E2 protective effects against colon cancer growth rely on E2-induced ERβ up-regulation [3]. In addition, ERα degradation is also required for the transcription of E2 responsive gene [7], [8]. As a whole, this evidence point to the control of ERα levels as a critical step in endocrine-dependent cell growth and, consequently, the identification of molecules that modulate these molecular circuitries is a demanding issue.

Endocrine disruptors (EDs) represent one of the best tools to evaluate the mechanisms underlying the control of ERα functions. Indeed, EDs represent a class of heterogeneous chemicals that are known to bind ERα and to interfere with many aspects of estrogen-dependent control of body homeostasis including the balance between cell growth/apoptosis; for that they have been also named xenoestrogens [9], [10]. Among other EDs, our research group has contributed to the definition of the actual paradigm that the plastic-derived food contaminant bisphenol A (BPA) and the plant-derived flavonoid naringenin (Nar) differently interfere with ERα-mediated signalling driving cancer cells to different functional outcomes. In particular, in ERα transiently transfected HeLa cells, BPA and Nar concentrations that completely saturate ERα (i.e., 10^−5^ M and 10^−6^ M, respectively) [2], [11], [12] induce cell proliferation and cell death, respectively. On the contrary, both molecules act as E2 mimetic on ERα-mediated ERE-containing gene transcription. Interestingly, 10^−6^ M Nar concentration is compatible with the concentration achieved in human blood after the ingestion of a meal rich in Nar and 10^−5^ M BPA is a sub-toxic concentration of this food contaminant [2].

This contrasting evidence on cell proliferation raises the question on the role of BPA and Nar on the modulation of ERα content alone or in combination with E2. To this purpose, we used the ERα-containing ductal carcinoma (MCF-7) cells to determine the effects of BPA and Nar on ERα protein and mRNA intracellular levels.

## Materials and Methods

### Cell Culture and Reagents

Human ductal carcinoma cells (MCF-7) and ER devoid human cervix carcinoma cells (HeLa) were grown as previously reported [4]. 17β-estradiol, cycloheximide (CHX), DMEM (with and without phenol red), charcoal-stripped fetal calf serum were purchased from Sigma-Aldrich (St. Louis, MO). Bradford protein assay was obtained from Bio-Rad (Hercules, CA). Antibodies against ERα (D12 mouse), against ubiquitin (P4D1 mouse) were obtained from Santa Cruz Biotechnology (Santa Cruz, CA); vinculin antibody was purchased from Sigma-Aldrich (St. Louis, MO). Anti-phospho-p38, anti-p38, anti-ERα Ser118 antibodies were purchased from Cell Signalling Technology Inc. (Beverly, MA). CDP-Star, chemiluminescence reagent for Western blot was obtained from PerkinElmer. p38/MAPK inhibitor, SB 203,580 (SB) and the 26S proteasome inhibitor MG132 were purchased by Calbiochem (San Diego, CA). All the other products were from Sigma-Aldrich. Analytical- or reagent-grade products, without further purification, were used.

### Biochemical Assays

Cells were grown in 1% charcoal-stripped fetal calf serum medium for 24 hrs and then stimulated with E2 at the indicated time points; where indicated, inhibitors (SB, MG132 and CHX) were added 1 h before E2 administration. Unless otherwise indicated, cell were treated with vehicle (DMSO, for Nar and BPA; ethanol for E2), E2 (10^−8^ M), Nar (10^−6^ M) or BPA (10^−5^ M). After treatment, cells were harvested with trypsin, centrifuged, stained with the trypan blue solution, and counted as previously described [13]. Protein extraction, biochemical assays and Western blots were performed as previously described [4].

### Plasmids, Transient Transfection and Luciferase Assay

The reporter plasmid 3×ERE TATA, the pcDNA flag-ER and the pcDNA flag-ERα S118A were previously described (4). HeLa cells were grown to 70% confluence and then transfected using lipofectamine reagent according to the manufacturer’s instructions. Three hours after transfection, the medium was changed, and 24 h after, the cells were serum starved for 24 h and then stimulated with E2 for 24 h. The cell lysis procedure as well as the subsequent measurement of luciferase gene expression was performed using the luciferase kit according to the manufacturer’s instructions with an PerkinElmer Life and Analytical Sciences (Bad Wildbad, Germany) luminometer as previously described [4].

### RNA Isolation and qPCR Analyses

The primers for human presenelin 2 (*pS2/TIFF*), cathepsin D (*CatD*), progesterone receptor (*PR*) and glyceraldehyde 3-phosphate dehydrogenase (*GAPDH*) were already described; Primers for human estrogen receptor α (*ERα*) 5′-GTGCCTGGCTAGAGATCCTG-3′ (forward) and 5′-AGAGACTTCAGGGTGCTGGA-3′ (reverse) were designed and used in RT-qPCR experiments as previously [4].

### Statistical Analyses

A statistical analysis was performed using the ANOVA test with the InStat version 3 software system (GraphPad Software Inc., San Diego, CA). Densitometric analyses were performed using the freeware software Image J by quantifying the band intensity of the protein of interest respect to the relative loading control band (*i.e.,* vinculin) intensity. All figures show representative blots. In all analyses, *p* values <0.01 were considered significant, but for densitometric analyses, *p* was <0.05. *p* values <0.001 were considered significant for growth curves analysis. Data are means of three independent experiments +/− SD.

## Results

### Bisphenol-A (BPA) and Naringenin (Nar) Impact of on ERα Activities in MCF-7 Cells

**Figure 1:**
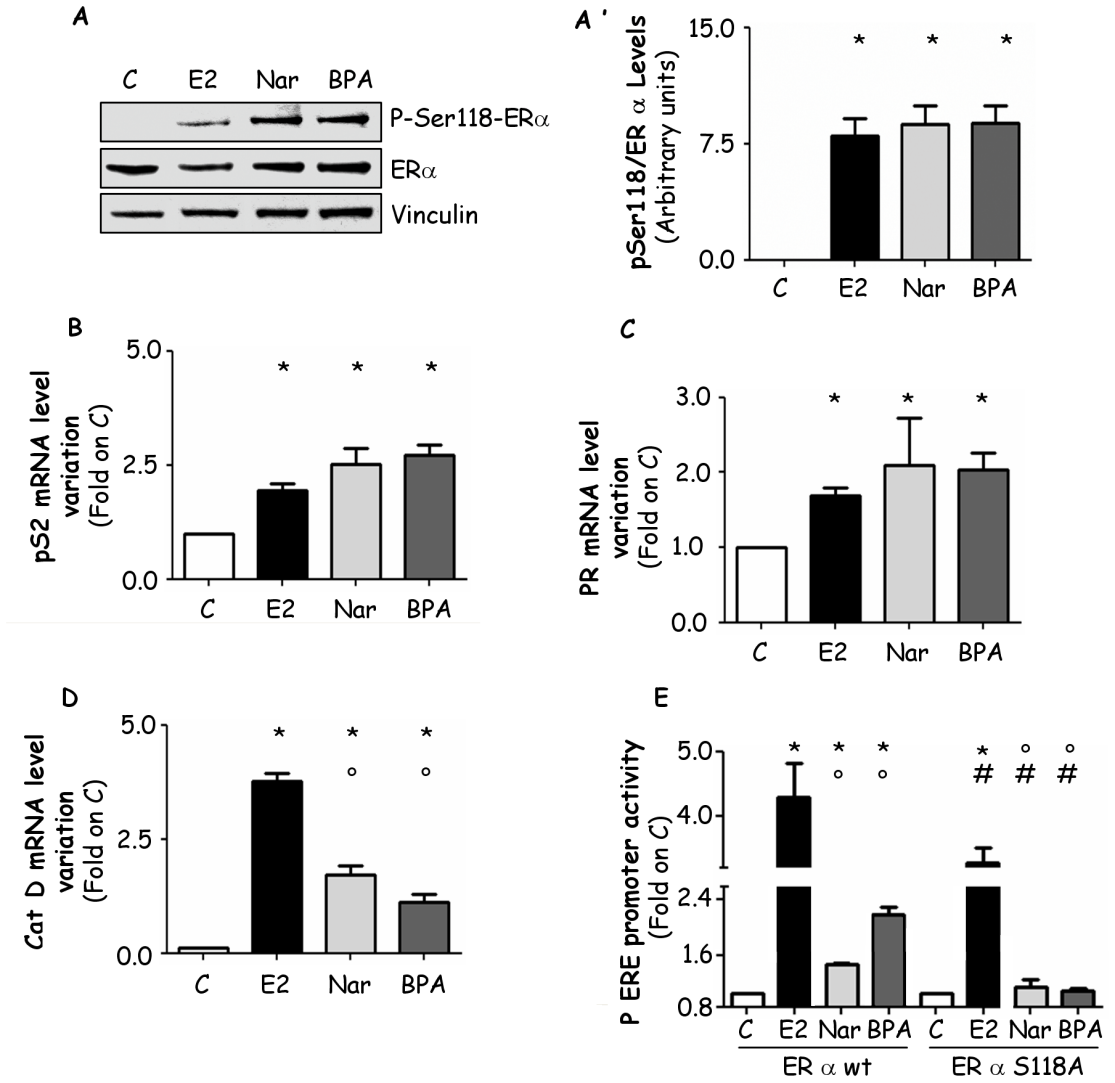
Effect of BPA and Nar on ERα activities. (Panel A) Western blot analysis of ERα S118 phosphorylation in MCF-7 cells treated with vehicle (C), E2, Nar or BPA for 2 hrs and relative densitometry. The same filter was re-probed with anti-ERα antibody. Loading control was performed by evaluating vinculin expression levels. * indicates significant differences with respect to the control sample. RT-qPCR analysis of presenelin 2 (pS2/TIFF) (panel B), progesterone receptor (PR) (panel C) and cathepsin D (CatD) (panel D) mRNA expression in MCF-7 cells treated with E2, Nar or BPA for 24 hrs. * indicates significant differences with respect to the control sample; ° indicates significant differences with respect to the E2-treated samples. (Panel E) HeLa cells were transfected with ERα wild type or with ERα S118A mutant together with a plasmid containing 3×ERE-TATA-luciferase construct. Cells were treated with vehicle E2, Nar or BPA for 24 hrs and the promoter activity was evaluated as described in material and method section. * indicates significant differences with respect to the control sample; ° indicates significant differences with respect to the E2-treated samples; # indicates significant differences with respect to the correspondent wild type samples.

**Figure 2:**
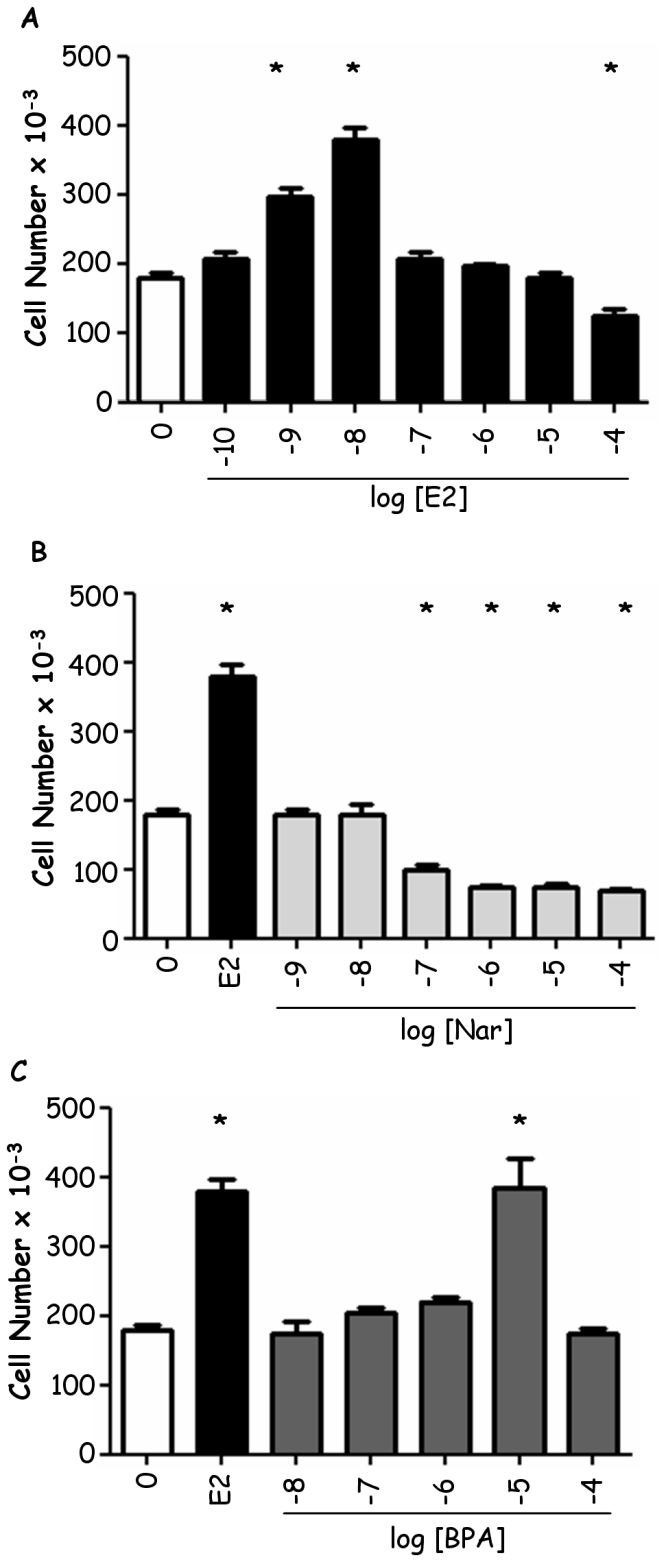
Effect of BPA and Nar on MCF-7 growth. MCF-7 cells were counted after 24 hrs treatment with vehicle (0), or different concentration of either E2 (panel A) or BPA (panel B) or Nar (panel C). * indicates significant differences with respect to the control sample.

The ERα phosphorylation in the serine (Ser) 118 residue has been recognized as an important step in the regulation of E2-induced ERα activities [8], [14]. This prompted us to evaluate if BPA and Nar could mimic this E2 regulation. Figure 1A shows that 2 hrs after E2 treatment the total ERα levels is slightly decreased; however, E2 increased by 7 fold the amount of ERα Ser118 phosphorylation with respect to the control. Intriguingly, BPA or Nar stimulation similarly to E2 increased the amount of Ser118 phosphorylation on ERα suggesting an E2 mimetic mechanism for both the EDs tested. Moreover, the ability of BPA and Nar to trigger the ERα transcriptional activity has been studied by evaluating the transcription of typical E2:ERα target genes containing a canonical ERE in the promoter [4] coding for: presenelin 2 (pS2/TIFF), progesterone receptor (PR), and cathepsin D (CatD). RT-qPCR analyses indicated that 24 hrs of (10^−5^ M) BPA or (10^−6^ M) Nar treatment, as E2 (10^−8^ M), increase the mRNA levels of pS2/TIFF and PR (Fig. 1B and 1C). Both EDs also increased CatD mRNA levels even if with a different efficiency than E2 (Fig. 1D). Interestingly, all tested ERα ligands needed an intact Ser118 site to exert their transcriptional effects. In fact, the transient transfection of HeLa cells, devoid of any ERs, with the ERα S118A mutant reduced the ability of E2 (about 40% ±10 with respect to ERα wild type) as previously reported [4] and abrogated the ability of Nar and BPA to activate the 3×ERE-TATA promoter (Fig. 1E). Finally, we evaluate the EDs impact on MCF-7 growth, a well known E2:ERα-dependent functional outcome. As reported in other cell lines [11], [15] BPA and E2 increased cell number with a maximum effect at 10^−8^ M and 10^−5^ M, respectively (Fig. 2A and 2B), whereas Nar from 10^−7^ M to 10^−4^ M significantly decreased the number of MCF-7 cells (Fig. 2C). As a whole these data indicate that even if all ERα ligands trigger receptor phosphorylation and transcriptional activity, BPA and Nar drive MCF-7 cells to a divergent proliferative outcome.

### Effect of BPA and Nar on ERα Level in MCF-7 Cells

**Figure 3:**
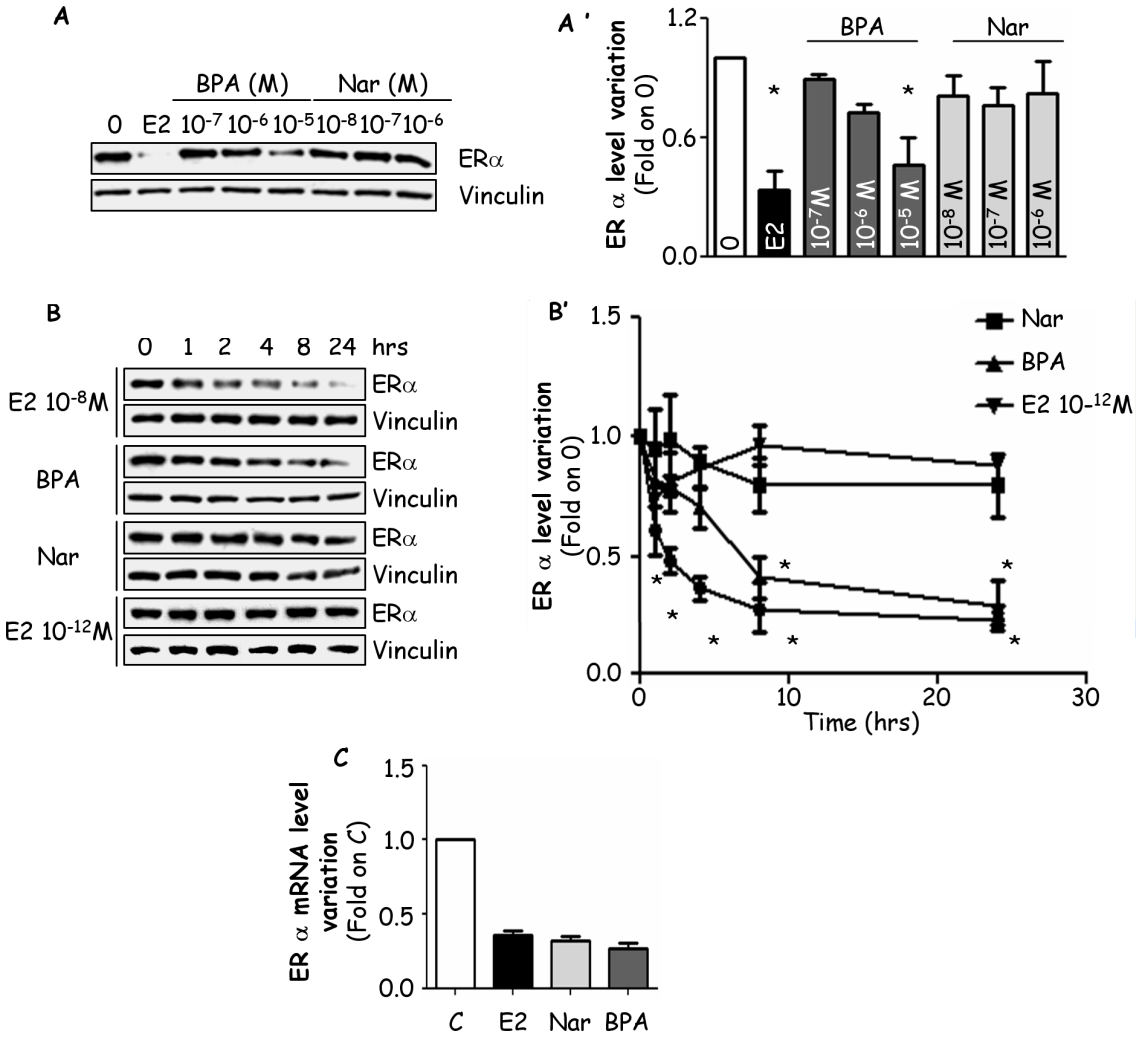
Effect of BPA and Nar on ERα level and expression. (Panel A) Western blot and (panel A’) relative densitometric analyses of ERα cellular levels in MCF-7 cells treated with vehicle (0) E2, Nar or BPA at the indicated doses for 24 hrs. (Panel B) Western blot and (panel B’) relative densitometric analysis of ERα cellular levels in MCF-7 cells treated with vehicle (0), E2 (10^−8^ or 10^−12^ M), Nar (10^−6^ M) or BPA (10^−5^ M) at different time points. (Panel C) RT-qPCR analysis of ERα mRNA expression in MCF-7 cells treated with vehicle (C), E2, Nar or BPA for 24 hrs. * indicates significant differences with respect to the control sample.

As expected, 24 hrs after E2 treatment ERα protein levels were reduced by 60% (Fig. 3A and 3A’). Similarly, a dose-dependent reduction in ERα intracellular levels were observed in cells exposed to BPA, while none of the Nar concentrations significantly affected the ERα content in MCF-7 cells (Fig. 3A and 3A’). A more detailed time course of BPA and Nar effect in MCF-7 cells confirmed that E2 (10^−8^ M) rapidly (2 hrs) induces ERα degradation while more time (i.e., 4 hrs) is necessary to BPA to halve ERα levels (Fig. 3B and 3B’). However, E2 (10^−8^ M) or BPA administration reduced ERα protein content to similar levels after 24 hrs of treatment (Fig. 3B and 3B’). Conversely, no significant changes in ERα protein content were detected when MCF-7 cells were treated with Nar or E2 (10^−12^ M) at all the tested time points (Fig. 3B and 3B’). RT-qPCR analyses (Fig. 3C) showed that the ERα mRNA content is reduced 24 hrs after E2 and BPA treatment; surprisingly, a similar reduction of ERα mRNA content was also found 24 hrs after Nar treatment (Fig. 3C).

**Figure 4:**
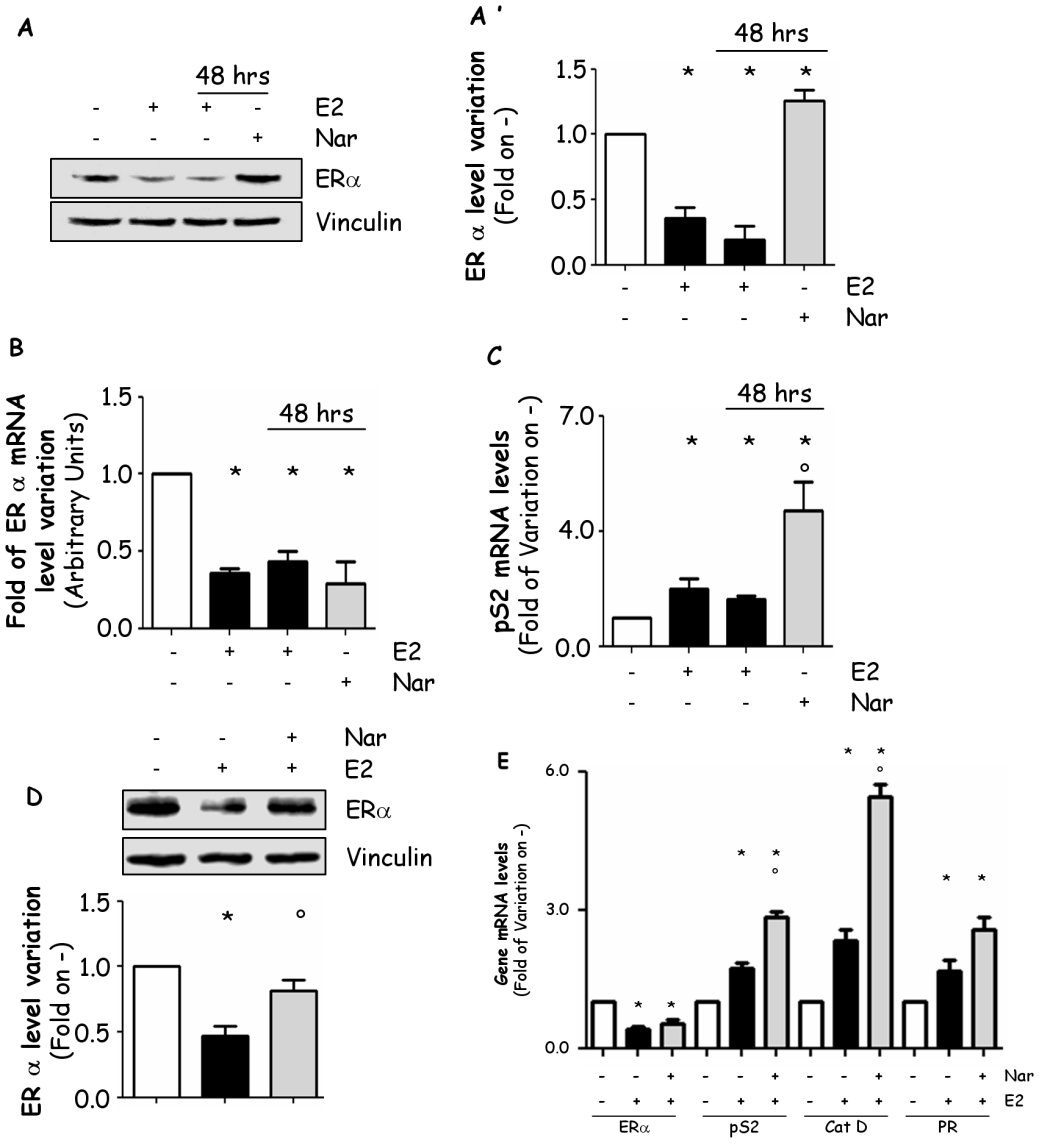
Long time Nar impact on ERα expression and transcriptional activity. (Panel A) Western blot and (panel A’) relative densitometric analysis of ERα cellular levels in MCF-7 cells treated with vehicle (−), E2 or Nar for 24 or 48 hours. RT-qPCR analysis of ERα (panel B) and presenelin 2 (pS2/TIFF) (panel C) mRNA expressions in MCF-7 cells treated with E2 or Nar for 24 or 48 hours. * indicates significant differences with respect to the control sample. (Panel D) Western blot analysis of ERα cellular levels and (panel E) RT-qPCR analysis of ERα, presenelin 2 (pS2/TIFF), progesterone receptor (PR) and cathepsin D (CatD) mRNA expression in MCF-7 cells treated with E2 for 24 hours both in the presence and in the absence of 24 hours pre-treatment with Nar. * indicates significant differences with respect to the control sample; ° indicates significant differences with respect to the corresponding E2 samples.

We next evaluated the Nar ability to control ERα expression by treating MCF-7 cells persistently (48 hrs). Figures 4A and 4A’ show that in MCF-7 cells the reduction in ERα cellular levels could be detected after 48 hrs of E2 stimulation. On the contrary, 48 hrs Nar increased ERα cellular content with respect to control-treated cells (Fig. 4A and 4A’). Moreover, RT-qPCR analysis confirmed that in MCF-7 cells 48 hrs of E2 or Nar treatment reduces ERα mRNA levels (Fig. 4B). Remarkably, a significantly higher level of the ERE-containing pS2/TIFF mRNA was detected when MCF-7 cells were stimulated 48 hrs with Nar than under E2 stimulation (Fig. 4C).

These data demonstrate that Nar-induced ERα accumulation could result in increased ERα transcriptional activity. In order to verify this possibility, we set up an experimental protocol where ERα intracellular levels and either ERα, pS2/TIFF, CatD or PR mRNA content were assayed in MCF-7 cells pre-treated for 24 hrs with Nar before additional 24 hrs of E2 co-administration. Under these conditions, we observed not only that Nar prevented the E2-induced reduction ERα intracellular levels (Fig. 4D) but also that the pS2/TIFF and CatD mRNA content was significantly higher in Nar-treated cells than in those where E2 was administrated (Fig. 4E). Interestingly, Nar pre-treatment in MCF-7 cells did not significantly affect ERα and PR mRNA levels (Fig. 4E).

This evidence strongly demonstrates that BPA, like E2, determines ERα down-regulation in 24 hrs, while Nar increases ERα cellular content up to 48 hrs modifying, consequently, ERα gene transcription.

### Effect of BPA and Nar on ERα Degradation

**Figure 5:**
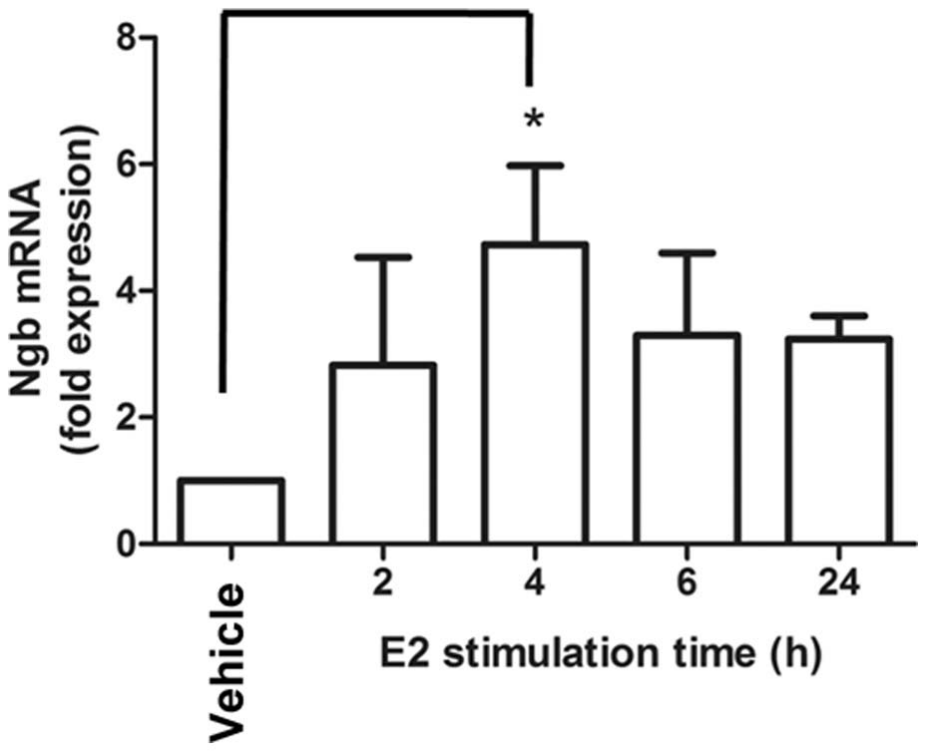
Mechanism of BPA- and Nar-dependent control of ERα degradation. Western blot analysis of ERα (panels A–C) or ubiquitin (panel C) cellular levels in MCF-7 cells treated with vehicle (−), E2, Nar or BPA for 24 hours in the presence of 60 min pre-treatment with cycloheximide (CHX) 1 µg/ml before ligand administration (panel A); or with E2, Nar, and CHX (1 µg/ml) at the indicated time points (panel B); or with E2 or BPA for 24 hours in the presence of 60 min pre-treatment with the 26S proteasome inhibitor Mg132 (1 µg/ml) before ligand administration (panel C). * indicates significant differences with respect to the control sample; ° indicates significant differences with respect to the corresponding non stimulated samples.

We used the protein-biosynthesis inhibitor cycloheximide (CHX) as a tool to study the effect of E2, BPA and Nar on the receptor degradation without the contribution of the neo-synthesized ERα pool (*i.e.,* pre-formend ERα). Twenty four hrs of E2, BPA or CHX treatment induced a significant reduction in total ERα cellular content while Nar did not affect it (Fig. 5A). When co-stimulation experiments where performed, E2 and BPA increased the effect of CHX on ERα breakdown and Nar treatment reduced the CHX-induced ERα degradation (Fig. 5A), thus suggesting that BPA, as E2, triggers proteolytic ERα degradation while Nar could induce an ERα intracellular accumulation.

Prompted by these results, we investigated the Nar effect on the pre-formed ERα cellular pool. Time-course analysis confirmed that prolonged (24–48 hrs) Nar treatment was able to reduce the CHX-dependent decay of ERα cellular content while E2 further increased it (Fig. 5B). In parallel, we also evaluated if BPA-dependent ERα degradation was ascribable, as in the case of E2, to the action of the 26S proteasome [7]. Pre-treatment of MCF-7 cells with the 26S proteasome inhibitor Mg132 blocked the 24 hrs E2- and BPA-induced ERα reduction in intracellular levels (Fig. 5C). As expected, the inhibition of the 26S proteasome induced an increase in the total amount of ubiquitinated proteins (Fig. 5C). These data further sustain that Nar affects ERα intracellular content differently than E2 and BPA.

### Role of p38/MAPK Pathway on the E2- and Nar-mediated Control of ERα Cellular Levels

**Figure 6:**
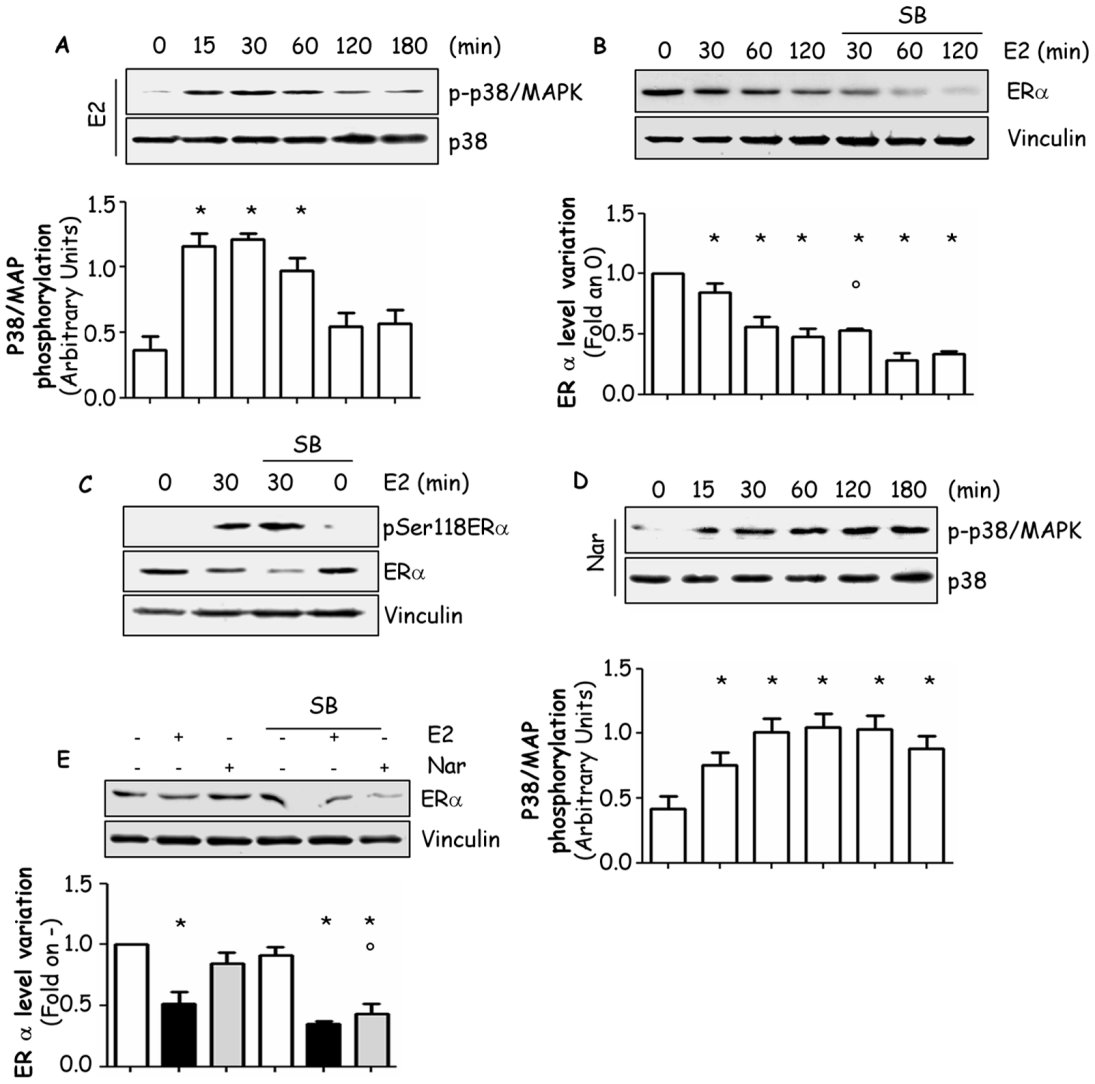
Involvement of p38/MAPK in E2- and Nar- control of ERα intracellular levels. Western blot analysis of p38/MAPK phosphorylation in MCF-7 cells treated with vehicle (0), E2 (panel A) or Nar (panel D) at different time points. The filter was re-probed with anti-p38. Western blot analysis of ERα cellular levels in MCF-7 cells treated with vehicle (0, -), E2 at different time points (panel B) or with E2 or Nar (panel E) for 2 hours. Western blot analysis of ERα S118 phosphorylation in MCF-7 cells treated with vehicle (0) or E2 for 30 min. The same filter was re-probed with anti-ERα antibody. Loading control was performed by evaluating vinculin expression levels (panel C). Where indicated, cells were treated for 60 min with the p38/MAPK inhibitor SB 203,580 (SB) (1 µM). Inhibitor alone was administrated for 3 hours. * indicates significant differences with respect to the relative control sample; ° indicates significant differences with respect to the corresponding non stimulated samples.

We recently demonstrated that ERα extra-nuclear signalling protects the receptor from E2-induced down-modulation [4]. In particular, the activity of phosphatidyl inositol 3 kinase (PI3K) but not of extracellular regulated kinase (ERK) is involved on E2 effects to the receptor down-regulation [4]. Moreover, we extensively reported in several cell lines that Nar is a specific antagonist of ERα-dependent rapid signals, in fact, Nar does not activate any of those signalling pathways [15], [16]. Conversely, this flavonoid rapidly and persistently activates p38/MAPK [17]. Thus, it is plausible that the rapid and prolonged activation of p38/MAPK triggered by Nar could shield ERα from breakdown in MCF-7 cells. As expected [17], E2 rapidly (15–30 min) triggers p38/MAPK phosphorylation, which was reduced after 2 hrs of E2 administration (Fig. 6A). Furthermore, incubation of MCF-7 cells with the p38/MAPK inhibitor SB 203,580 (SB) increased the time-dependent E2-triggered reduction in the ERα cellular amount with a statistically significant effect occurring 30 min after E2 stimulation (Fig. 6B and 6D) but did not influence the E2-induced ERα phosphorylation on the Ser residue 118 (Fig. 6C). Nar evoked a rapid (15 min) and persistent (3 hrs) increase in p38/MAPK phosphorylation in MCF-7 cells (Fig. 6D). Remarkably, in the presence of p38/MAPK inhibitor Nar induced the reduction of ERα cellular levels. However, no effect of SB on basal receptor levels (Fig. 6E) was detected. These data indicates that the activation of the p38/MAPK pathway defends ERα from ligand-mediated degradation.

## Discussion

One of the fundamental principles of endocrinology is that hormones exert their physiological actions through receptors [10]. From this tenet derives that the hormone-regulated receptor levels is one of the fundamental mechanism(s) by which a hormone regulates the functional endpoints in target cells. A long exposure (i.e., more than 24 hrs) or hormone doses which saturate receptors down-regulate receptor levels, leading to a decrease in sensitivity of target cells. However, E2-induced down-regulation of ERα is completed 2–4 hrs after 10^−8^ M hormone stimulation in breast cancer cell lines in parallel with the appearance of E2:ERα-mediated gene transcription and E2 physiological effects which result in a typical “bell-shaped” or “inverted U” curve (Fig. 1). Thus, it is expected that xenoestrogens will replicate or impair this hormone characteristics modulating the ERα cellular content.

Here we used BPA and Nar, two xenoestrogen prototypes, which differently modulate ERα activities acting, respectively, as E2 mimetic and as anti-E2:ERα extra-nuclear signalling [2], [15]. As predictable by ERα binding characteristics [11], [12], both BPA and Nar activate ERα by triggering Ser118 phosphorylation and transcriptional activities on ERE-contained E2 target genes. However, only BPA mimics E2 in inducing breast cancer cell proliferation, whereas Nar reduces cell number in a dose-dependent manner. Intriguingly, the dose response curve of BPA is reminiscent of the ‘bell shaped’ curve induced by E2, contrarily, Nar stimulation results in a sigmoid dose response curve which suggest that this plant-derived ED could exert a different regulation of receptor levels.

ERα protein levels are regulated by a dynamic balance between ERα synthesis and breakdown [7]. Particularly, the native ERα protein level is under the control of the 26S proteasome and the ERα polyubiquitination is the signal to activate receptor degradation. Exposure to E2 results in a hormone-dependent reduction in the total ERα content trough the 26S proteasome-dependent degradation of the neo-synthesized ERα and of the E2-activated ERα (*i.e.,* pre-formed receptor) [7]. Interestingly, hormone-dependent down-regulation that leads to rapid and extensive loss of receptor is characteristic of other nuclear steroid receptors, including human progesterone receptors (PRs). In particular, in breast cancer cells, it has been demonstrated that PR is phosphorylated by ERK in Ser294 and degraded by a 26S proteasome-mediated pathway 6 hrs after treatment with progestin [18]. These results indicate that steroid hormones evoke surprising similar mechanisms to trigger receptor down-regulation. Analysis of the modality by which BPA and Nar affect ERα protein intracellular content reveals that BPA mimics the E2 effects in inducing the 26S proteasome-dependent ERα degradation. On the contrary, Nar induces the receptor accumulation by blocking ERα proteolytic degradation of the pre-formed receptor as demonstrated by the Nar effect in the presence of the protein-biosynthesis inhibitor cycloheximide or by chronic treatment of MCF-7 cells with Nar.

Although we did not evaluate the role of BPA and Nar on the neo-synthesized ERα, we further studied the effects of these EDs on ERα mRNA levels. E2-induced ERα degradation occurs with a parallel reduction in the ERα mRNA levels [7]. Interestingly, all of the ERα ligands mimic E2 in controlling the ERα mRNA levels (Fig. 2B, 5B and 6B), thus sustaining the concept that BPA and Nar modulate ERα expression through two different mechanisms. The different effect of Nar in increasing ERα protein levels and in reducing receptor mRNA levels unveils a complex post-transcriptional Nar-dependent regulation of ERα content. ERα mRNA synthesis can be regulated by ERα promoter methylation and by different ERα-specific microRNAs (miRNAs) [19]. Thus, our evidence could be reconciled by taking into account the miRNA-dependent regulation of ERα expression. In this respect, although Nar-dependent miRNA regulation has never been reported, recent data indicate that flavonoids (*e.g.,* genistein, daidzedin) could function as miRNA regulators [20]. Additionally, Nar-dependent epigenetic modifications of the ERα promoters could occur and affect ERα expression [5]. All together, these discoveries demonstrate that BPA controls ERα expression by triggering an ERα-based signalling similar to the one induced by the E2-activated receptor.

On the contrary, Nar engagement to ERα hijacks the receptor intracellular signalling that impact on the Nar-dependent modulation of ERα expression. In line with this assumption, the mechanism by which Nar induces the accumulation in ERα intracellular levels requires the persistent activation of the p38/MAPK. Signalling modulation of ERα intracellular levels appears to be dependent on the activation of the E2-evoked ERα extra-nuclear kinase cascades [4]. In particular, we have recently reported that the E2-dependent activation of the PI3K/AKT but not of the ERK/MAPK pathway protects ERα from the E2-induced proteolytic breakdown [4] and, more recently, p38/MAPK has been implicated in the regulation of ERα turnover [21]. Our results confirm that E2 determines the rapid and transient activation of the p38/MAPK pathway in ERα-containing cells also in breast cancer cells and further demonstrate that the E2-activated p38/MAPK pathway is involved in the regulation of ERα intracellular levels. Thus, in addition to the PI3K/AKT pathway [4], the p38/MAPK pathway contributes to the E2-dependent control of ERα cellular levels. Accordingly, Nar does not affect PI3K/AKT and ERK/MAPK pathway activation in the presence of ERα while it induces *via* ERα the persistent activation of the p38/MAPK cascade [2] which is involved in Nar-dependent control of ERα intracellular levels. Indeed, in the presence of the pharmacological inhibition of the p38/MAPK (*i.e.,* SB treatment), Nar acquires the ability to trigger ERα degradation. Thus, we can conclude that the persistent activation of the p38/MAPK triggered by Nar protects ERα from the proteolytic breakdown.

ERα phosphorylation on the Ser118 residue is necessary for the extra-nuclear signalling-dependent protection of ERα from E2-induced degradation [4]. However, we found that p38/MAPK inhibition does not prevent E2-induced ERα Ser118 phosphorylation (Fig. 6C). In addition, Nar or BPA treatment still induces the receptor phosphorylation event (Fig. 1D). Interestingly, other ERα residues (*i.e.,* serine 294 and threonine 311) are the reported targets of ERα p38/MAPK-dependent phosphorylation [21], [22]. Thus, additional phosphorylation sites may be required for ERα protection from proteolytic degradation. Nonetheless, in agreement with the concept that ERα Ser118 phosphorylation controls full ERα transcriptional activity [14], BPA- and Nar-dependent ERα Ser118 phosphorylation is required for ERE-containing ERα target gene transcription (*i.e.,* pS2/TIFF, CatD, PR as well as artificial ERE-containing construct).

More importantly, we also found that the Nar-dependent accumulation of ERα results in an increased receptor transcriptional activity and that, upon Nar stimulation, E2 looses its capacity to regulate ERα turnover and to physiologically control ERα gene transcription. Indeed, the Nar-dependent blockade of E2-induced ERα down-modulation has the consequence to enhance pS2/TIFF and CatD transcriptional activity. These discoveries indicate that in a cellular context exposed to Nar the absolute physiological receptor response or the one in response to E2 is changed because of deregulated receptor expression. Thus, Nar modulation of ERα cellular content could further affect the E2-dependent regulation of specific cellular processes (*i.e.,* proliferation) leading to scenarios that strongly diverge from the physiological ones.

In conclusion, we demonstrate here that exposure to EDs drastically modifies ERα expression, which, in turn, conveys an altered response to the E2-dependent physiological one. Numerous clinical and *in vitro* studies suggest that the alteration (*i.e.,* reduction) of ERα expression is an important step in the development and progression of E2-related disease including breast cancer [19]. Although it is known that BPA could elicit *in vivo* effects in a dose lower than that used in our experiments [23], the results reported here indicate that 10^−5^ M BPA, a non-toxic dose which saturate ERα [11], induces cancer cell proliferation through ERα down-regulation. On the other hand, ERα-dependent Nar anti-proliferative actions ([15] and present results) could rely on its ability to prevent the E2-dependent reduction in ERα down-regulation strongly confirming the preventive effects elicited by Nar on E2-dependent cancers [12]. However, the information reported here together with that showing xenoestrogens as agonist (i.e., flavonoid) or antagonist (i.e., BPA) of E2 in the presence of ERβ subtype [24], [25] strongly sustain that xenoestrogens modify the estrogen milieu impairing the hormone subtle balance of proliferation/apoptosis obtained by the maintenance of ERs levels [5], [26]. Thus, women, but even men who express estrogen receptors, may be considered highly susceptible population with an increased risk of breast cancers after BPA exposures.
