# Correction: Xenoestrogens Alter Estrogen Receptor (ER) α Intracellular Levels

**DOI:** 10.1371/journal.pone.0099379

**Published:** 2014-05-30

**Authors:** The PLOS ONE Staff

The corresponding authors are not noted in the HTML version of the paper. Dr. Filippo Acconcia (filippo.acconcia@uniroma3.it) and Dr. Maria Marino (maria.marino@uniroma3.it) are the corresponding authors.

Figure 5 is incorrect. Please see the corrected Figure 5 here.

**Figure 5:**
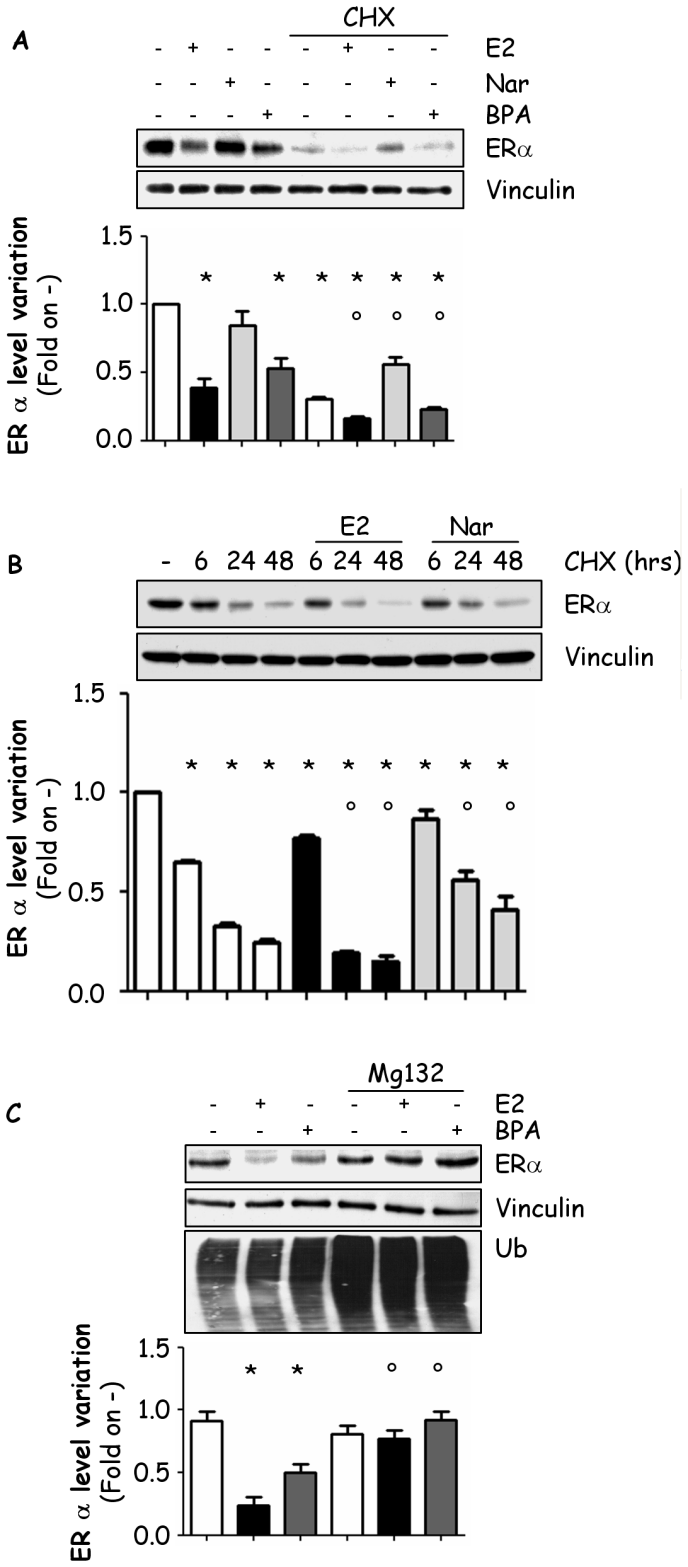
Mechanism of BPA- and Nar-dependent control of ERα degradation. Western blot analysis of ERα (panels A-C) or ubiquitin (panel C) cellular levels in MCF-7 cells treated with vehicle (-), E2, Nar or BPA for 24 hours in the presence of 60 min pre-treatment with cycloheximide (CHX) 1 µg/ml before ligand administration (panel A); or with E2, Nar, and CHX (1 µg/ml) at the indicated time points (panel B); or with E2 or BPA for 24 hours in the presence of 60 min pre-treatment with the 26S proteasome inhibitor Mg132 (1 µg/ml) before ligand administration (panel C). * indicates significant differences with respect to the control sample; ° indicates significant differences with respect to the corresponding non stimulated samples.
